# Thermal Denaturation and Aggregation of Myosin Subfragment 1 Isoforms with Different Essential Light Chains

**DOI:** 10.3390/ijms11114194

**Published:** 2010-10-27

**Authors:** Denis I. Markov, Eugene O. Zubov, Olga P. Nikolaeva, Boris I. Kurganov, Dmitrii I. Levitsky

**Affiliations:** 1 A.N. Bach Institute of Biochemistry, Russian Academy of Sciences, Leninsky prosp. 33, 119071, Moscow, Russia; E-Mails: dm.marik.230185@gmail.com (D.I.M.); ezubov@perevozki.ru (E.O.Z.); boris@kurganov.com (B.I.K.); 2 A.N. Belozersky Institute of Physico-Chemical Biology, Moscow State University, 119992, Moscow, Russia; E-Mail: olga.nikolaeva6@mail.ru (O.P.N.)

**Keywords:** myosin subfragment 1, thermal denaturation, heat-induced aggregation, differential scanning calorimetry, dynamic light scattering

## Abstract

We compared thermally induced denaturation and aggregation of two isoforms of the isolated myosin head (myosin subfragment 1, S1) containing different “essential” (or “alkali”) light chains, A1 or A2. We applied differential scanning calorimetry (DSC) to investigate the domain structure of these two S1 isoforms. For this purpose, a special calorimetric approach was developed to analyze the DSC profiles of irreversibly denaturing multidomain proteins. Using this approach, we revealed two calorimetric domains in the S1 molecule, the more thermostable domain denaturing in two steps. Comparing the DSC data with temperature dependences of intrinsic fluorescence parameters and S1 ATPase inactivation, we have identified these two calorimetric domains as motor domain and regulatory domain of the myosin head, the motor domain being more thermostable. Some difference between the two S1 isoforms was only revealed by DSC in thermal denaturation of the regulatory domain. We also applied dynamic light scattering (DLS) to analyze the aggregation of S1 isoforms induced by their thermal denaturation. We have found no appreciable difference between these S1 isoforms in their aggregation properties under ionic strength conditions close to those in the muscle fiber (in the presence of 100 mM KCl). Under these conditions kinetics of this process was independent of protein concentration, and the aggregation rate was limited by irreversible denaturation of the S1 motor domain.

## 1. Introduction

Cyclic association-dissociation of myosin and actin, coupled with myosin-catalyzed ATP hydrolysis, is the most essential event in muscle contraction. The globular head of myosin, called subfragment 1 (S1), is responsible for the force generation during contraction. It consists of a globular motor (or catalytic) domain that contains the sites responsible for the ATP hydrolysis and actin binding, and a neck region with a long *α*-helix extending from the globular part of the head and forming a complex with the essential and regulatory light chains [1]. It has been proposed that this light chain binding domain, also called regulatory domain, acts as a semi-rigid “lever arm” to amplify and transmit the conformational changes occurring in the ATP and actin binding sites of S1 [2,3].

S1 prepared by chymotryptic digestion of skeletal muscle myosin lacks the regulatory light chain but does contain the essential light chain, also called alkali light chain [4]. In fast skeletal muscle myosin there are two kinds of alkali light chains, A1 and A2. These light chains are nearly identical except for first 41 additional amino acid residues of the A1 light chain, containing repeated Ala-Pro sequence and two pairs of lysine residues located near the *N*-terminus [5]. The functional significance of this *N*-terminal extension of the A1 light chain is currently under investigation. Since there are two kinds of alkali light chains, A1 and A2, chymotryptic S1 preparation is a mixture of two isoforms containing either only A1 or only A2 (S1(A1) and S1(A2), respectively). These two S1 isoforms can be separated by using ion-exchange chromatography [4], and they are commonly used for comparative studies of the function of A1 and A2 light chains and for elucidation of the role of the *N*-terminal extension in A1. It was shown that the affinity of S1(A1) for actin at low ionic strength is significantly higher than that of S1(A2) [6,7], and this is due to direct interaction of the *N*-terminal extension of the A1 light chain with actin [8–11]. It should be noted, however, that the interaction of the *N*-terminal portion of the A1 light chain with actin decreased markedly as the ionic strength was increased up to 120 mM [7].

Another interesting property of the *N*-terminal extension of the A1 light chain is its interaction with the globular motor domain of the myosin head [12–14]. Recent studies have shown direct binding of the *N*-terminal extension of A1 to the SH3 domain located near the *N*-terminus of the heavy chain (residues 35–80) and predicted a significant role of this binding in the actin-myosin interaction [14]. Note that all these experiments were performed at relatively low ionic strength (up to 25–50 mM).

Previous studies showed a dramatic difference between two S1 isoforms in their heat-induced aggregation measured at low ionic strength: S1(A1) aggregated at much lower temperature than S1(A2) [15,16]. This implies that the *N*-terminal extension of A1, due to its semi-rigid, antenna-like structure [17], can be involved not only in intramolecular interaction, but also into intermolecular interactions with motor domains of other S1 molecules. It seems possible that these interactions may induce some conformational changes in the S1 motor domain, which can be expressed in changes of its thermal denaturation.

In the present work, we applied differential scanning calorimetry (DSC) for a detailed comparative analysis of thermal denaturation of two S1 isoforms, S1(A1) and S1(A2), and, in particular, to investigate the domain structure of these S1 isoforms. For this purpose, a new calorimetric approach was specially developed to analyze the DSC profiles of irreversibly denaturing multidomain proteins (see Section 2). Using this approach, we have revealed two calorimetric domains in the S1 molecule, the more thermostable domain denaturing in two steps. We also applied special approaches to identify these calorimetric domains, *i.e.*, to reveal their correspondence to structural domains of the S1 molecule. Furthermore, we applied dynamic light scattering (DLS) to analyze aggregation of the two S1 isoforms induced by their thermal denaturation and to elucidate the mechanism of this aggregation. The experiments were performed at physiological ionic strength (100 mM KCl), and the results were compared with those obtained at low ionic strength both in earlier studies [15,16] and in the present work. We have found no appreciable difference between S1(A1) and S1(A2) in their aggregation under conditions used (100 mM KCl). It has been shown that kinetics of this process is independent of the protein concentration and the aggregation rate is limited by irreversible denaturation of the motor domain. Some difference between the two S1 isoforms was only revealed by DSC, independently of the ionic strength conditions, in the thermal denaturation of the regulatory domain. Thus, the interaction of the *N*-terminal extension of the A1 light chain with the motor domain seems to be too weak to produce any effect on its thermal denaturation even at low ionic strength.

## 2. New Approach to Analysis of Irreversible Denaturation of Multidomain Proteins

### 2.1. Theory

In this section a new method of the analysis of thermograms for multidomain proteins undergoing irreversible denaturation is proposed. Thermograms for such proteins represent the sum of separate peaks corresponding to individual calorimetric domains (since calorimetric domain is a part of protein molecule that denatures cooperatively and independently from the other parts). Up to date, there has not been proposed any general analytical solution for the problem of the decomposition of thermograms into individual domains with the simultaneous determination of the kinetic parameters for these domains.

For the sake of simplicity it is assumed that denaturation of each individual calorimetric domain is described by the one-stage model of irreversible denaturation:

(1) $$N\;\overset{k}{\to }\;D$$

where *N* and *D* are native and denatured states of the protein and *k* is the rate constant of the first order (or pseudo-first order) reaction. Further, it is assumed that the dependence of the rate constant *k* on temperature follows the Arrhenius law:

(2) $$k=\text{exp}\left[\frac{E_{\alpha }}{R}\cdot \left(\frac{1}{T*}-\frac{1}{T}\right)\right]$$

where *E*_a_ is the experimental energy of activation, *R* is the universal gas constant, *T* is the absolute temperature, and T^*^ is the temperature at which *k* = 1 min^−1^ (the dimension of *k* in this formula is min^−1^). The kinetics of denaturation is described by the equation:

(3) $$\frac{dx}{dt}=-kx$$

where *x* is the portion of the native protein (at *t* = 0 the *x* value is equal to unity). If the temperature is varied with a constant rate (*dT/dt* ≡ ν), Equation 3 acquires the following form:

(4) $$\frac{dx}{dT}=-\frac{k}{\nu }\cdot x$$

Integration of this equation gives the expression for the portion of the native form as a function of temperature:

(5) $$x(T)=\text{exp}\left[-\frac{1}{\nu }\int_{T_{0}}^{T}\text{exp}\left(\frac{E_{\alpha }}{R}\left(\frac{1}{T*}-\frac{1}{T}\right)\right)dT\right]$$

If one assume that enthalpy of denaturation is independent of temperature, the following expression may be obtained for the excess heat capacity $C_{\text{p}}^{\text{ex}}$:

(6) $$C_{\text{p}}^{\text{ex}}(T)=\frac{dQ^{\text{ex}}}{dT}=-\Delta H\cdot \frac{dx}{dT}=\frac{\Delta H}{\nu }\cdot k(T)\cdot x(T)$$

where *Q*^ex^ is the excess heat absorbed at denaturation. This dependence may be presented in an expended form:

(7) $$C_{\text{p}}^{\text{ex}}(T)=\frac{\Delta H}{\nu }\cdot \text{exp}\left[\frac{E_{\alpha }}{R}\cdot \left(\frac{1}{T*}-\frac{1}{T}\right)\right]\cdot \text{exp}\left[-\frac{1}{\nu }\int_{T_{0}}^{T}\text{exp}\left(\frac{E_{\alpha }}{R}\left(\frac{1}{T*}-\frac{1}{T}\right)\right)dT\right]$$

Kurganov *et al*. [18] elaborated the following method of the determination of parameters *E_a_* and T* from the experimental thermogram. From Equations 4 and 6 we can obtain the expression for the rate constant *k* as a function of temperature:

(8) $$k(T)=\frac{\nu \cdot C_{\text{p}}^{\text{ex}}(T)}{\Delta H\cdot x(T)}=\frac{\nu \cdot C_{\text{p}}^{\text{ex}}(T)}{\Delta H-Q^{\text{ex}}(T)}=\frac{\nu \cdot C_{\text{p}}^{\text{ex}}(T)}{\int_{0}^{+\infty }C_{\text{p}}^{\text{ex}}(T)dT-\int_{0}^{T}C_{\text{p}}^{\text{ex}}(T)dT}=\frac{\nu \cdot C_{\text{p}}^{\text{ex}}(T)}{\int_{T}^{+\infty }C_{\text{p}}^{\text{ex}}(T)dT}$$

Substituting of *k* into Equation 8, carrying out a logarithmic operation and multiplying of both parts of the equation by *T* gives:

(9) $$\frac{E_{\alpha }}{R\cdot T*}\cdot T-\frac{E_{\alpha }}{R}=T\cdot \left(\text{ln}(\nu )+\text{ln}(C_{\text{p}}^{\text{ex}}(T))-\text{ln}\int_{T}^{+\infty }C_{\text{p}}^{\text{ex}}(T)dT\right)\equiv W(T)$$

As can be seen, on the one hand, the function *W*(*T*) is completely determined by the experimental curve of the excess heat capacity. On the other hand, in the case of the fulfillment of the one-stage scheme of irreversible denaturation this function is linear with respect to temperature. Thus, the parameters of the Arrhenius equation can be easily estimated through the coefficients of the linear regression.

The thermogram $C_{\text{p}}^{\text{ex}}(T)$ for the multidomain protein consisting of *N* calorimetric domains is an algebraic sum of the signals of the individual calorimetric domains $C_{\text{p},\;\;i}^{\text{ex}}(T)$:

(10) $$C_{\text{p}}^{\text{ex}}(T)=\sum_{i=1}^{N}C_{\text{p},\;\;i}^{\text{ex}}(T)$$

The experimental calorimetric enthalpy of denaturation is expressed as follows:

(11) $$\Delta \text{H}=\sum_{i=1}^{N}\Delta H_{i}$$

where Δ*H_i_* is the enthalpy of denaturation of *i*-th domain. Preliminary incubation of such a protein at a certain constant temperature over some period of time just before the calorimetric experiment (denoted below in the text as *annealing*) results generally in partial denaturation of each domain. The shape of calorimetric peak for each domain remains unchanged, however the decrease in the registered enthalpy of the transition occurs: Δ*H_i_*, _ann_ = *x_i_* · Δ*H_i_*, where *x_i_* is a portion of *i*-th domain remaining in the native form after the annealing (0 *< x_i_* *<* 1). It follows herefrom that $C_{\text{p},\;i,\;\text{ann}}^{\text{ex}}(T)=x_{i}\cdot C_{\text{p},\;\;i}^{\text{ex}}(T)$. Thus, thermogram of the whole protein is expressed as follows:

(12) $$C_{\text{p},\;\text{ann}}^{\text{ex}}(T)=\sum_{i=1}^{N}x_{i}\cdot C_{\text{p},\;i}^{\text{ex}}(T)$$

Solution of Equation 3 at a constant temperature within the incubation time interval *t*_ann_ gives us the *x_i_* value: *x_i_* = exp[–*k_i_*(*T*_ann_) · *t*_ann_], where *k_i_*(*T*_ann_) is the rate constant for denaturation of *i*-th domain at the temperature *T*_ann_ calculated from the Arrhenius equation (2). Thus, *x_i_* is the function depending on *t*_ann_ and *T*_ann_ and parametrically depending on *E_a,i_* and $T_{i}^{*}$ (*i.e.*, parameters of the Arrhenius equation for *i*-th domain).

If the calorimetric experiment with preliminary incubation (annealing) of the protein preparation has been carried out (*N* −1) times at various temperatures over various incubation time intervals, we obtain the following system of *N* equations which are linear with respect to $C_{\text{p},\;\;i}^{\text{ex}}(T)$:

(13) $$\left\{C_{\text{p},\;\text{ann},\;\text{j}}^{\text{ex}}(T)=\sum_{i=1}^{N}x_{i,\;j}\cdot C_{\text{p},i}^{\text{ex}}(T)\right\},\;\;\;j=1,2,\ldots N$$

where *j* corresponds to the number of the experiment. The case when *j* = *N* corresponds to the experiment without preliminary incubation (*i.e.*, for all values of *i* the identity *x_i, N_* ≡ 1 is valid).

If domains are numbered in order of increasing thermostability, the following condition holds at *j* ≠ *N: x*_1,_ *_j_* *< x*_2,_ *_j_* < . . . < *x_N, j_* . From the theoretical point of view, if only one of parameters *E*_a_ and T^*^ for (*i* – 1)-th and *i*-th calorimetric domains has different values (otherwise these domains should be considered as a single domain), it is possible to select the temperature and time of annealing, at which *x_i_*_–1,_ *_j_* ≈ 0 (in practice, the inequality *x_i_*_–1,_ *_j_* < 0.01 should be met) and *x_i_*_,_ *_j_* ≫ 0. From the predicate of the numeration of domains follows that under these conditions of incubation 1^st^, 2^nd^, . . . , (*i* – 2)-th domains are also annealed, *i.e.*, *x*_1,_ *_j_*, *x*_2,_ *_j_*, . . . , *x_i_*_–2,_ *_j_* ≈ 0. Thus, it is possible to select the set of annealing conditions that makes the system of equations (13) transformed into the triangle form:

(14) $$\left\{ \begin{array}{ll} C_{\text{p},\;\;\text{ann},\;\;1}^{\text{ex}}(T) & =0\;\cdot \;C_{\text{p},\;\;1}^{\text{ex}}(T)+0\;\cdot \;C_{\text{p},\;\;2}^{\text{ex}}(T)+\ldots +0\;\cdot \;C_{\text{p},\;\;N-1}^{\text{ex}}(T)+x_{N,1}\cdot C_{\text{p},\;\;N}^{\text{ex}}(T)\\ C_{\text{p},\;\;\text{ann},\;2}^{\text{ex}}(T) & =0\;\cdot \;C_{\text{p},\;\;1}^{\text{ex}}(T)+0\;\cdot \;C_{\text{p},\;\;2}^{\text{ex}}(T)+\ldots +x_{N-1,2}\cdot C_{\text{p},\;\;N-1}^{\text{ex}}(T)+x_{N,2}\cdot C_{\text{p},\;\;N}^{\text{ex}}(T)\\ & \ldots \\ C_{\text{p},\;\;\text{ann},\;\;N-1}^{\text{ex}}(T) & =0\cdot C_{\text{p},\;\;1}^{\text{ex}}(T)+x_{2,\;N-1}\cdot C_{\text{p},\;\;2}^{\text{ex}}(T)+\ldots \\ & \;\;\;\;\;\;\;\;\;\;\;\;\;\;\;\;\;\;\;\;\;\;\;\;\;\;\;\;\;\;\;\;\;\;\;\;\;\;\;\;\;\;\;\;\;\;\;\;\;\;\;\;\;\;\;\;\;\;\;\;\;\;\;\;\;\;\;\;\;\;\;\;\;\;\;+x_{N-1,\;N-1}\cdot C_{\text{p},\;\;N-1}^{\text{ex}}(T)+x_{N,\;\;N-1}\cdot C_{\text{p},\;\;N}^{\text{ex}}(T)\\ C_{\text{p}}^{\text{ex}}(T) & =1\cdot C_{\text{p},\;\;1}^{\text{ex}}(T)+1\cdot C_{\text{p},\;\;2}^{\text{ex}}(T)+\ldots +1\cdot C_{\text{p},\;N-\;1}^{\text{ex}}(T)+1\cdot C_{\text{p},\;\;N}^{\text{ex}}(T) \end{array}\right.$$

The left parts of the system contain the functions of excess heat capacity obtained directly from the experiment, whereas the right parts of the system are the linear combinations of the functions to be found. Since the determinant of the system differs from zero, the system has unambiguous solution. To find this solution, we have to select properly the corresponding annealing parameters, namely the temperature and duration of each incubation. A rigorous solution of this problem is lacking. However, the following approach may be used to obtain a rough solution.

Evidently, in the part of thermogram corresponding to high temperatures it is possible to select a region where all domains except for the most thermostable one are already completely denatured. In this part of the thermogram $C_{\text{p}}^{\text{ex}}(T)=C_{\text{p},\;\;N}^{\text{ex}}(T)$, *i.e.*, the thermogram of the multidomain protein coincides with the thermogram of the most thermostable domain. In this region of temperatures the function *W*(*T*) (see Equation 9) is linear. This enables parameters *E_a_* and T^*^ for the most thermostable domain to be estimated. With a knowledge of parameters *E_a_* and T^*^, we can calculate *x_N_*(*T*) and *k_N_*(*T*). The optimization of the whole thermogram by thermogram corresponding to the most thermostable domain in the coordinates $C_{\text{p}}^{\text{ex}}(T)$ vs (*k_N_* (*T*) · *x_N_*(*T*)*/*ν) (see Equation 6) in this region of temperatures using the least squares method allows parameter Δ*H_N_* to be determined.

Based on these considerations one can propose the following recursive method for the estimation of parameters of calorimetric domains for multidomain protein. After obtaining of $C_{\text{p}}^{\text{ex}}(T)$ from the DSC data we calculate the function *W*(*T*) and conclude that the thermogram is not described by the one-stage model of irreversible denaturation. Using *W*(*T*) we estimate parameters of the most thermostable domain: *E_a, N_*, $T_{N}^{*}$ and Δ*H_N_* . With a knowledge of these parameters, we simulate $C_{\text{p},\;\;N}^{\text{ex}}(T)$ and subtract it from the whole thermogram of protein. Then this procedure is repeated for the function $\left(C_{\text{p}}^{\text{ex}}(T)-C_{\text{p},\;\;N}^{\text{ex}}(T)\right)$, which is the overall thermogram of all domains except for the most thermostable domain. As a result we obtain parameters for (*N* – 1)-th domain, simulate function $C_{\text{p},\;\;\;N-1}^{\text{ex}}(T)$ and subtract it from the function ( $C_{\text{p}}^{\text{ex}}(T)-C_{\text{p},\;\;N}^{\text{ex}}(T)$). By repeating this procedure we estimate parameters for (*N* – 2)-th domain, (*N* – 3)-th domain and so on down to first calorimetric domain.

However, when determining parameters of *N*-th domain, we should take into account that the error will be rather high because the region of temperatures where the function *W*(*T*) is linear is small in comparison with the temperature interval for the whole thermogram. Besides, to determine the corresponding parameters, the high-temperature branch of the DSC profile is used where $C_{\text{p}}^{\text{ex}}(T)$ is close to zero and the signal-to-noise ratio is rather small. The function *W*(*T*) in this region is even noisier since it contains the term ln $C_{\text{p}}^{\text{ex}}(T)$. All the above is valid also for parameters of (*N*–1)-th domain. Moreover, the additional error, connected with the inexact subtraction of simulated function $C_{\text{p},\;\;N}^{\text{ex}}(T)$, arises. This results in accumulation of the error. To weaken such an effect, the parameters, which are calculated at each step for the corresponding domain by the above-mentioned method, should be optimized in the temperature interval where they were determined. Here optimization is regarded as a minimization in the parameter space (in the least square sense) of the residuals between simulated (using the estimated parameters of the domain) and experimental values of heat capacity. Though even in that case the calculated parameters should be considered as a null approximation only, they allow selecting the time and temperature for the annealing procedures. It is significant that the above-mentioned approach enables the number of thermal transitions in a multidomain protein to be determined with a high accuracy.

When the corresponding annealing procedures have been carried out and all thermograms have been obtained, the multidimensional non-linear optimization in the parameter space may be used for final calculation of parameters *E_a_*, T^*^ and Δ*H* for all calorimetric domains.

### 2.2. Resolving Power of the Method. Selection of the Annealing Conditions

The method is based on annealing of 1st, 2nd, . . . , (*i*–1)-th domains, where *i* is a number of domain in the order of increasing thermostability. This provides a means for the determination of parameters of *i*-th domain. From the theoretical point of view, if the values of only one of parameters *E_a_* or T^*^ for two domains are non-identical, the conditions of annealing may be selected at which *x_i_*_–1_ ≈ 0 and *x_i_* *>* 0. However, the acceptable values of *x_i_*_−1_ and *x_i_* are the following: *x_i_*_−1_ *<* 0.01 and *x_i_* *>* 0.1. Otherwise the determination of parameters of *i*-th domain becomes impossible. The acceptable time of annealing is at least 10 min. If time of annealing is less than 10 min, delayed heating of the sample to the temperature of incubation and following cooling inevitably lead to an unacceptably large error. In other words, the following conditions should be satisfied:

(15) $$\left\{ \begin{array}{l} \text{exp}[-k_{i}(T_{\text{ann}})\cdot t_{\text{ann}}]>0.1\\ \text{exp}[-k_{i-1}(T_{\text{ann}})\cdot t_{\text{ann}}]<0.01\\ t_{\text{ann}}>10 \end{array}\right.\;\;\;\;\text{or}\;\;\;\left\{ \begin{array}{l} k_{i}(T_{\text{ann}})\cdot t_{\text{ann}}<\text{ln}10\\ k_{i-1}(T_{\text{ann}})\cdot t_{\text{ann}}>2\cdot \text{ln}\;10\\ t_{\text{ann}}>10 \end{array}\right.\;\;\;$$

If the values of the activation energy for two domains are about 300 kJ/mole and the values of parameter T^*^ are about 320 K, after the substituting of the Arrhenius equation in the expressions for the corresponding rate constants and solving the system of inequalities (15) we obtain the following limitations. For the case of identical T^*^ values the minimum reasonable difference between parameters *E_a_* is ∼150 kJ/mole. For the case of identical values of the activation energy the difference $T_{i}^{*}-T_{i-1}^{*}$ should not be less than 2 K.

Taking into account the above-mentioned requirements to the annealing conditions, we can propose the following method for the selection of *T*_ann_ and *t*_ann_. For definiteness we fix the ratio *x_i_*_−1_/*x_i_* ≡ *A* (with the requirements for *x_i_*_−1_ and *x_i_* we obtain that *A <* 0.1). Finally we come to the system of inequalities:

(16) $$\left\{ \begin{array}{l} \frac{x_{i-1}(T_{\text{ann}},\;t_{\text{ann}})}{x_{i}(T_{\text{ann}},\;t_{\text{ann}})}=A\\ x_{i}(T_{\text{ann}},\;t_{\text{ann}})\geq \text{min}(x_{i})=0.1\\ t_{\text{ann}}\geq 10 \end{array}\right.$$

The solution relative to *t*_ann_ is a curve *t*_ann_ = *f*(*T*_ann_) established by the conditions:

(17) $$\left\{ \begin{array}{l} t_{\text{ann}}=\frac{\text{ln}(A)}{k_{i}(T_{\text{ann}})-k_{i-1}(T_{\text{ann}})}\\ t_{\text{ann}}\leq \frac{-\text{ln}(\text{min}(x_{i}))}{k_{i}(T_{\text{ann}})}\\ t_{\text{ann}}\geq 10 \end{array}\right.$$

The coordinates of each point on this curve give the appropriate conditions for annealing.

## 3. Results and Discussion

### 3.1. Thermal Denaturation of Myosin S1 Isoforms Studied by DSC

**Figure 1.**
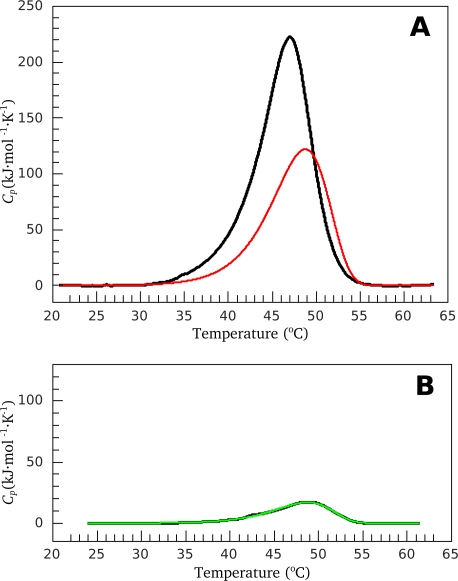
Temperature dependence of $C_{\text{p}}^{\text{ex}}$ for S1(A1) in the presence of 100 mM KCl. Checking of the validity of the three-domain model. (**A**) DSC profile for the original protein preparation (black) and the simulated curve for the most thermostable calorimetric domain (red); (**B**) DSC profile for the same S1(A1) preparation obtained after annealing at 46 °C for 15 min (black) and its fitting in the framework of the one-stage irreversible model (green).

Let us consider how the above-described principles of DSC data analysis can be applied to S1(A1) thermal denaturation in the presence of 100 mM KCl. Figure 1A shows a DSC profile for the original protein preparation (black curve). Preliminary analysis of this thermogram allowed us to reveal three irreversible transitions. So, zero approach suggests existence of three independent calorimetric domains undergoing one-stage irreversible denaturation. Using our method, we calculated the annealing conditions (15 min at 46 °C) for elimination of all domains except the most thermostable one. Figure 1B shows the DSC profile of the annealed preparation. This profile is satisfactorily described by the one-stage scheme of irreversible denaturation, so the corresponding values of *E_a_*, T^*^ and Δ*H_trans_* (the area under the experimental $C_{\text{p}}^{\text{ex}}$ curve) parameters can be easily found. Taking into account the conditions of annealing, we can calculate the Δ*H* for this domain using equation:

(18) $$\Delta H=\Delta {\text{H}}_{trans}\cdot \text{exp}\left[t_{\text{ann}}\cdot \text{exp}\left(\frac{E_{\alpha }}{R}\cdot \left(\frac{1}{T*}-\frac{1}{T_{\text{ann}}}\right)\right)\right]$$

where *t*_ann_ and *T*_ann_ are the annealing time (in min) and temperature (in K), respectively. Knowing all three parameters of the calorimetric domain, we can simulate its thermogram. Figure 1A shows comparison of such a simulated thermogram of the most thermostable domain (red curve) with the thermogram of initial S1(A1) preparation obtained before the annealing procedure (black curve). Obviously, the thermogram of one of the calorimetric domains should be the part of the thermogram of the entire protein. It is seen from Figure 1A that this is not the case. Similar results were obtained for S1(A1) under low ionic strength conditions, as well as for S1(A2) independently of ionic strength (data not shown).

It was proposed from earlier DSC studies that the total heat sorption curve of S1 is composed of three independent calorimetric domains [16,19–21]. However, our results (Figure 1A) indicate that this assumption is incorrect and the S1 thermogram cannot be described as a sum of three independent calorimetric domains.

We have proposed somewhat more complicated model for the S1 thermal denaturation, which presumes two calorimetric domains in the S1 molecule. According to this model, the less thermostable domain (designated as domain I) undergoes one-stage irreversible denaturation, whereas thermal denaturation of more thermostable domain (calorimetric domain II) is described by two-stage scheme:

(19) $$N\overset{k_{1}}{\to }I\overset{k_{2}}{\to }D$$

where *N* and *D* correspond to native and denatured states of the protein and *I* is a kinetic intermediate state. First-order rate constants *k*_1_ and *k*_2_ obey the Arrhenius law (Equation 2), and both stages are accompanied by positive thermal effect (Δ*H*_1_, Δ*H*_2_ > 0). Correspondingly, the thermogram of such a domain can be described as follows

(20) $$C_{\text{p}}^{\text{ex}}(T)=\Delta H_{1}\cdot k_{1}(T)\cdot x_{N}(T)+\Delta H_{2}\cdot k_{2}(T)\cdot x_{I}(T)$$

where *x_N_* is the portion of the native state and *x_I_* is the portion of the kinetic intermediate state:

(21) $$x_{N}(T)=x_{N}^{0}\cdot \text{exp}\left(-\int_{T_{0}}^{T}\frac{k_{1}(T)}{\nu }dT\right)$$

(22) $$x_{I}(T)=\text{exp}\left(-\int_{T_{0}}^{T}\frac{k_{2}(T)}{\nu }dT\right)\cdot \left[x_{I}^{0}+\int_{T_{0}}^{T}\frac{k_{1}(T)\cdot x_{N}(T)}{\nu }\cdot \text{exp}\left(\int_{T_{0}}^{T}\frac{k_{2}(T)}{\nu }dT\right)\right]$$

$x_{N}^{0}$ and $x_{I}^{0}$ correspond to initial values of the portion of the native state and the kinetic intermediate state under conditions when *T* = *T*_0_. Obviously, before annealing, $x_{N}^{0}=1$ and $x_{I}^{0}=0$ (i.e., this domain is in its native state in all protein molecules). However, this is not the case for protein preparations which have undergone the annealing procedure. In this case, both $x_{N}^{0}$ and $x_{I}^{0}$ can be easily calculated by solving a system of differential equations corresponding to the model of thermal denaturation (19) and by finding parameters *k*_1_ and *k*_2_ from the Arrhenius equation (Equation 2). It is evident that, under some rather hard conditions of the annealing, we can obtain the protein preparation, in which $x_{N}^{0}\approx 0$ and $x_{I}^{0}\neq 0$. The thermogram for such type preparation can be described by the following equation:

(23) $$C_{\text{p}}^{\text{ex}}(T)\approx \Delta H_{2}\cdot k_{2}(T)\cdot x_{I}(T)\approx \Delta H_{2}\cdot k_{2}(T)\cdot x_{I}^{0}\cdot \text{exp}\left(-\int_{T_{0}}^{T}\frac{k_{2}(T)}{\nu }dT\right)$$

that corresponds to the thermogram of the calorimetric domain, which undergoes one-stage irreversible denaturation. The same situation can also take place, starting from some temperature *T* = *T*′_0_ on the DSC profile, for protein preparation that has not been subjected to annealing procedure. Therefore, when the above-proposed algorithm is applied for analysis of the high-temperature region of the S1 thermogram, we can erroneously consider the second stage of the thermal denaturation of the most thermostable S1 domain as independent calorimetric domain. This indirectly evidences the model of S1 thermal denaturation involving two calorimetric domains, one of which denatures in two stages thus yielding two thermal transitions. Since independent calorimetric domains often correspond to independent structural domains, two S1 calorimetric domains may reflect the thermal denaturation of the main S1 structural domains, the motor domain and the regulatory domain, and this may again indicate that the two-domain model of the S1 thermal denaturation is correct.

To find the parameters of the S1 calorimetric domains according to the above described two-domain model for the S1 thermal denaturation, we have used multidimensional nonlinear optimization in the space of these parameters. The following input data were simultaneously used for this analysis: the thermograms of native S1(A1) and S1(A2) preparations, the thermograms of these preparations subjected to two different annealing procedures (*i.e.*, to preheating for definite time at definite temperature), as well as the time and temperature of these annealing procedures. The parameters obtained by this way are presented in Table 1. Each thermal transition is characterized by calorimetric enthalpy (Δ*H*) and two parameters of the Arrhenius equation, namely, activation energy (*E_a_*) and special temperature (T^*^, *i.e.*, the temperature at which the rate constant is equal to 1 min^−1^).

**Figure 2.**
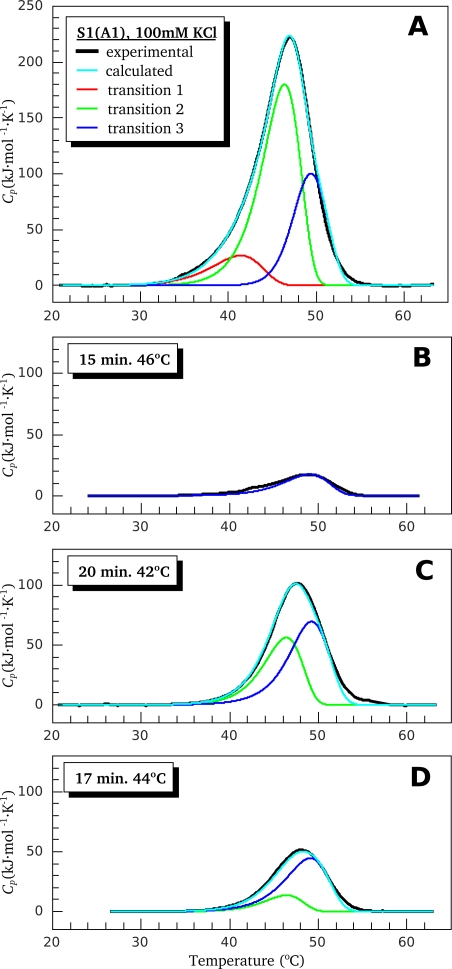
Analysis of the DSC data obtained for S1(A1) in the presence of 100 mM KCl in the framework of the two-domain model. Black curves correspond to the experimental data. (**A**) DSC profile for the original preparation; (**B** and **C**) DSC-profiles for the same preparation after annealing at 46 °C for 15 min and at 42 °C for 20 min, respectively. The results of the fitting are represented by the colored curves. Cyan corresponds to the full DSC profile. Red, green, and blue curves correspond to transitions 1, 2, and 3, respectively; (**D**) DSC profile for the S1(A1) preparation after annealing at 44 °C for 17 min. The colored curves were simulated using parameters given in Table 1.

Figure 2A–C shows the results of decomposition of S1(A1) thermograms, as measured in the presence of 100 mM KCl, into thermal transitions calculated from the above proposed model of S1 thermal denaturation using parameters given in Table 1. Note that thermal transitions 2 and 3 correspond to the first and the second stages of denaturation of the most thermostable calorimetric domain. To verify the proposed model and the calculated calorimetric parameters (Table 1), we have performed control annealing procedure by preheating the S1(A1) preparation for 17 min at 44 °C; however, the thermograms obtained from this experiment were not used in optimization procedure and, therefore, in calculation of the calorimetric parameters. Figure 2D represents the comparison of the experimentally obtained thermogram of S1(A1) subjected to the control annealing (black curve) with that simulated using calorimetric parameters given in Table 1 (cyan curve). Close similarity of both thermograms allows us to conclude that the proposed two-domain model is correct, as well as the calorimetric parameters calculated in the framework of this model.

**Figure 3.**
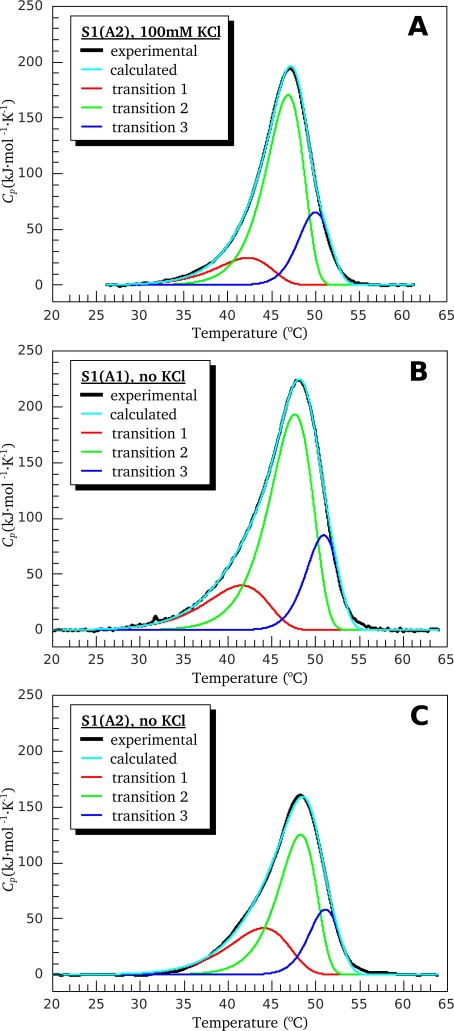
Analysis of the DSC data obtained for S1 isoforms in the framework of the two-domain model. (**A**) S1(A2) in the presence of 100 mM KCl; (**B**,**C**) S1(A1) and S1(A2) at low ionic strength, in the absence of KCl (20 mM Hepes, pH 7.3). Black curves correspond to the experimental data. The results of the fitting are represented by the colored curves. Cyan corresponds to the full DSC profile. Red, green, and blue curves correspond to transitions 1, 2, and 3, respectively. The colored curves were simulated using parameters given in Table 1.

Similar experiments were performed with S1(A1) under low ionic strength conditions, as well as with S1(A2) independently of ionic strength. Figure 3 shows the results of decomposition of DSC thermograms for all these preparations. Overall, these DSC results, together with the parameters presented in Table 1, indicate that both S1 isoforms, S1(A1) and S1(A2), denature similarly and independently of the ionic strength conditions. Noticeable differences in the thermal denaturation between the two S1 isoforms were only revealed in parameter T^*^ of the least thermostable calorimetric domain (transition 1 in Figures 2, 3 and domain I (or transition 1) in Table 1). This parameter is lower by 1.5–2.0 K in the case of S1(A1) in comparison with S1(A2) (Table 1).

**Figure 4.**
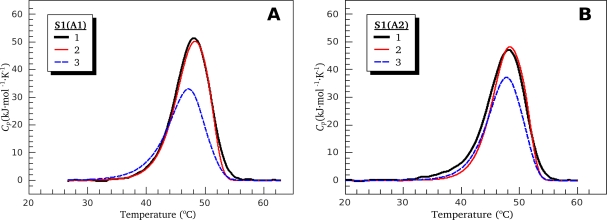
Comparison of the experimental DSC profiles (black curves) obtained after control annealing procedure (17 min at 44 °C) for S1(A1) (**A**) and S1(A2) (**B**) with simulated curves (red and blue). See the text for more details.

In order to confirm the difference between S1(A1) and S1(A2) in the T^*^ parameter of domain I, we have performed additional optimization procedure, as follows. All calorimetric parameters (except only T^*^ of domain I) were allowed to change and domains’ I T^*^ values were set equal to the average value among S1(A1) and S1(A2) (318.3 K under high ionic strength conditions) and frozen. The parameters obtained after such an optimization were used to simulate the DSC profiles of S1 isoforms after control annealing (17 min at 44 °C) (curves 3 on Figure 4), and these simulated DSC curves were compared with the experimentally obtained DSC profiles (curves 1 on Figure 4). Figure 4 also shows the DSC curves simulated using all the parameters given in Table 1 (curves 2). It is seen from Figure 4 that curves 2 almost completely coincide with experimentally obtained curves 1, whereas curves 3 simulated according to assumption of domains’ I T^*^ values equality are quite different from curves 1. Thus, we can conclude that this assumption is erroneous, and therefore the difference in domains’ I T^*^ values between S1 isoforms is reliable. This means that domain I is more thermostable in S1(A2) than in S1(A1). It should be noted that this effect is independent of ionic strength conditions as it is observed both at low ionic strength and in the presence of 100 mM KCl.

### 3.2. Thermally-Induced Changes in the Intrinsic Fluorescence of S1 Isoforms

**Figure 5.**
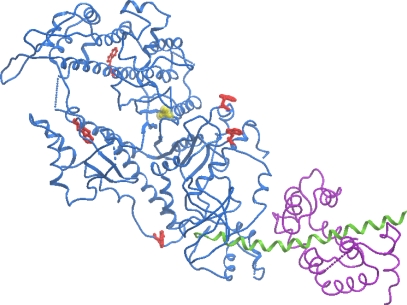
Three-dimensional structure of chymotryptic S1. Tryptophan residues are colored by red, nucleotide associated with ATPase site is colored by yellow.

To identify the S1 calorimetric domains (*i.e.*, to reveal their correspondence to S1 structural domains) and to verify the DSC results by another independent method, we have studied the temperature dependence of the intrinsic tryptophan fluorescence of S1 isoforms. There are five Trp residues in the S1 molecule [22], and all these residues are located in the S1 motor domain (Figure 5). Therefore, one can expect that the heat-induced changes of tryptophan fluorescence parameters will occur in the temperature region corresponding to thermal denaturation of the motor domain.

**Figure 6.**
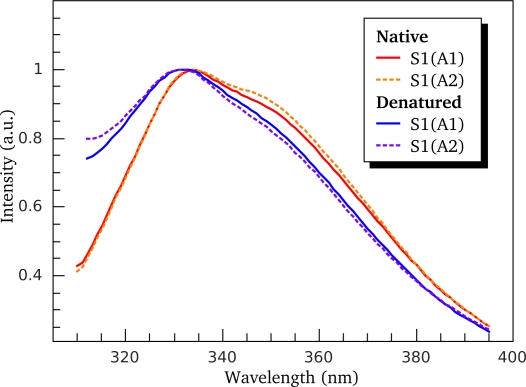
Normalized spectra of intrinsic tryptophan fluorescence of S1(A1) (solid line curves) and S1(A2) (dashed line curves) measured before (red curves) and after (blue curves) the heating of S1 isoforms up to 70 °C performed in the presence of 100 mM KCl with heating rate of 1 °C/min.

Figure 6 shows normalized tryptophan fluorescence spectra of S1(A1) and S1(A2) measured before (red curves) and after (blue curves) heating the S1 isoforms up to 70 °C. It is seen that heating results in the spectral shift towards shorter wavelengths. This effect was observed for the both S1 isoforms not only in the presence of 100 mM KCl (Figure 6) but also at low ionic strength (data not shown). In all the cases, this spectral shift was accompanied by an increase in parameter *A*

(24) $$A=\frac{I_{320}}{I_{365}}$$

(where *I*_320_ and *I*_365_ are fluorescence intensities at λ_em_ = 320 and 365 nm, respectively) from 1.05–1.07 to 1.30–1.35. This means that the environment of tryptophan residues (at least some of them) becomes more hydrophobic after irreversible thermal denaturation. The only explanation for this strange behavior of S1 is that its irreversible thermal denaturation cannot be simply considered as trivial unfolding. Apparently, we are still far from detailed understanding of the mechanism of irreversible thermal denaturation of proteins.

**Figure 7.**
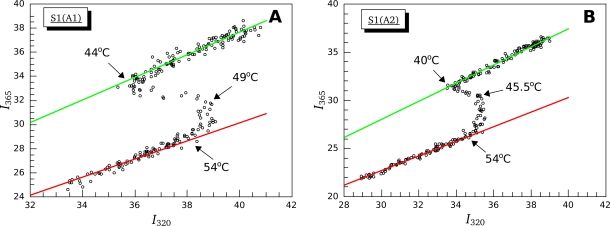
Parametric dependence of intensity of fluorescence at 365 nm on fluorescence intensity at 320 nm, characterizing the temperature-induced denaturation of S1(A1) (**A**) and S1(A2) (**B**). The variable parameter is the temperature. Fluorescence characteristics of native and completely denatured Trp-containing regions of S1 are represented by green and red lines, respectively. Values of fluorescence intensities are expressed in relative units. Both parametric curves were obtained from the heating of both S1 isoforms with constant rate of 1 °C/min in presence of 100 mM KCl.

For a more detailed analysis of the changes in the fluorescent properties of S1 isoforms induced by their thermal denaturation, we applied the method of parametric plots [23]. Figure 7 shows the dependence of fluorescence intensity at 365 nm on the intensity of fluorescence at 320 nm obtained at different temperatures. In this case, the fluorescence intensities at 320 and 365 nm were used as independent extensive characteristics, and the temperature was taken as a parameter. The linear parts of the plot extrapolated to zero correspond to the common thermal fluorescence quenching without any change in the protein structure. Extrapolations of such parts of the plot representing native and denatured states of Trp-containing regions are shown as green and red lines, respectively. The linear (quasi-linear) parts between these lines, which cannot be extrapolated to zero, correspond to conversion of one structural state into another state (only in the case of the system composed of two states with different microenvironment of tryptophan residues) [24]. It is clearly seen from Figure 7 that at least two transition processes take place for both S1 isoforms as two quasi-linear segments, which cannot be extrapolated to zero, are observed on the parametric plots. The first of them is located within the temperature region from 44 °C to 49 °C for S1(A1) (Figure 7A) and from 40 °C to 45.5 °C for S1(A2) (Figure 7B), and then it is substituted by the second segment, which lasts up to 54 °C for both S1 isoforms. It is important to note that both these segments are located in the temperature region corresponding to thermal transitions 2 and 3 revealed by DSC studies (Figures 2A and 3A). Furthermore, the end of the second segment (54 °C for both S1 isoforms) (Figure 7) coincides with the end of calorimetric transition 3 (Figures 2A and 3A). Thus, we can assert that in all the cases the temperature-induced changes of S1 fluorescent properties occur in two stages that approximately correspond to calorimetric transitions 2 and 3, *i.e.*, they correspond to the thermal denaturation of the most thermostable calorimetric domain, which denatures in two stages.

**Figure 8.**
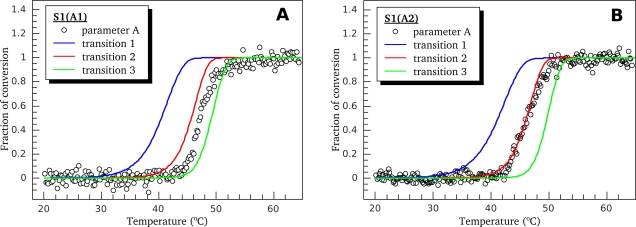
Temperature-induced changes of the S1(A1) (**A**) and S1(A2) (**B**) intrinsic fluorescence measured by the changes of the value of normalized parameter *A* (black circles) in comparison with fraction of conversion for calorimetric transitions 1, 2, and 3 obtained from DSC experiments (colored curves). Normalized parameter *A* was calculated according to Equation 25. Fraction of conversion of transition 2 was calculated as 1 – *x_N_* ; fraction of conversion of transition 3 was calculated as *x_D_*, where *x_N_* and *x_D_* are fractions of the states *N* and *D* of calorimetric domain II according to scheme (19).

It should be noted that detailed quantitative analysis of parametric dependences is rather difficult, mainly because of small number of experimental points in the region of transition processes. An increase of the number of points decreases the data accumulation time thus raising the noise. Another problem is imperfect correspondence between temperature scales of the instruments used, calorimeter and fluorimeter, which cannot be fully eliminated even by the calibration procedure. Extremely high sensitivity of non-linear models to even small errors can make the results of fluorescence data optimization incomparable with those obtained from the DSC data analysis. Therefore, to compare more precisely the temperature-induced changes in fluorescent properties of S1 isoforms with thermal denaturation of their calorimetric domains, we analyzed the temperature dependences of normalized parameter *A*

(25) $$A_{\text{norm}}=\frac{A-A_{\text{min}}}{A_{\text{max}}-A_{\text{min}}}$$

(where *A*_min_ and *A*_max_ represent minimal and maximal values of the parameter *A*, respectively) and compared the fraction of conversion from native to denatured state calculated using this fluorescent parameter with that obtained from the DSC data for calorimetric domains of S1 (Figure 8). It is clearly seen that, for both S1 isoforms, the temperature-induced changes of parameter *A* occur within the temperature region corresponding to calorimetric transitions 2 and 3, *i.e.*, in the region of thermal denaturation of the second calorimetric domain, while no changes of parameter *A* are observed in the region of thermal denaturation of calorimetric domain I (thermal transition 1) (Figure 8). Taking into account the fact that all tryptophan residues are localized in the motor domain of the S1 molecule (Figure 5), our results allow to identify the second calorimetric domain (involving thermal transitions 2 and 3) as the motor domain and calorimetric domain I—as the regulatory domain.

### 3.3. Heat-Induced Inactivation of S1 ATPase

**Figure 9.**
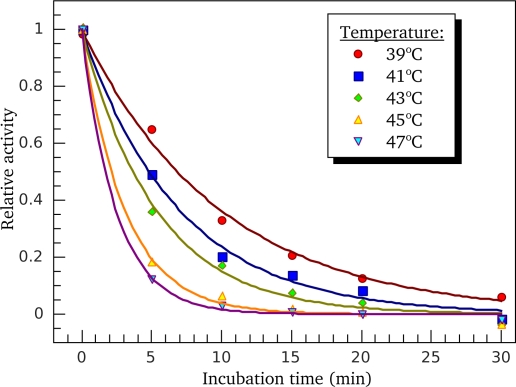
Typical kinetics of heat-induced inactivation of S1 K^+^-EDTA-ATPase obtained at different temperatures for S1(A2) preparation in presence of 100 mM KCl. Protein concentration was 0.5 mg/mL. The experimental data are shown by colored points, and solid curves represent the results of their mono-exponential decay fitting.

**Figure 10.**
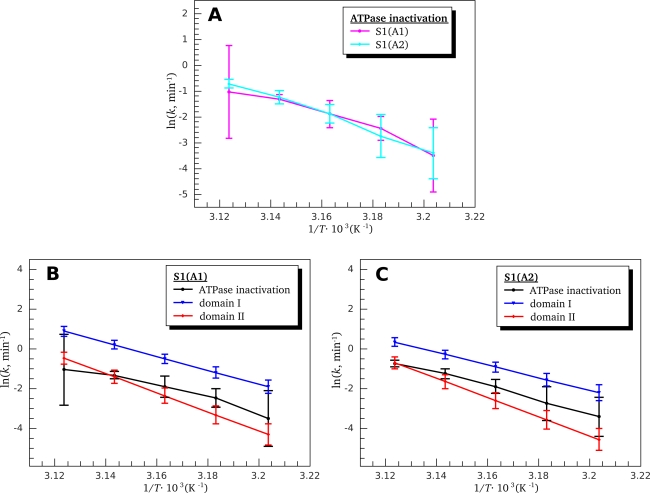
Temperature dependences of the first-order rate constants calculated for heat-induced inactivation of K^+^-EDTA-ATPase of S1 isoforms. (**A**) Comparison of the inactivation rate constants for S1(A1) (magenta) and S1(A2) (cyan). (**B**, **C**) Comparison of the ATPase inactivation rate constants (black lines) for S1(A1) (**B**) and S1(A2) (**C**) with the rate constants calculated for thermal denaturation of calorimetric domain I (blue lines) and for the first stage of denaturation of calorimetric domain II (red lines). The rate constants for thermal denaturation of calorimetric domains were calculated using parameters given in Table 1.

For further identification of the calorimetric domains of S1 isoforms we studied the temperature-induced inactivation of their ATPase. Figure 9 shows typical kinetic curves of heat-induced inactivation of S1 ATPase obtained at different temperatures. It is seen that these dependences are well described by exponential curves, *i.e.*, they correspond to the first-order (pseudo-first-order) reactions, thus allowing us to calculate from these experimental data the rate constants for S1 ATPase inactivation at different temperatures. These rate constants calculated for S1(A1) and S1(A2) were almost identical within the error limits (Figure 10A). We also have calculated the rate constants for thermal denaturation of calorimetric domain I and the first stage of denaturation of calorimetric domain II (transition 2) using the parameters *E_a_* and T^*^ obtained from the DSC studies (Table 1). Comparison of these rate constants with those calculated for ATPase inactivation of S1 isoforms is presented in Figure 10B, C. It is clearly seen from this comparison that the rate constants of ATPase inactivation are very close to those calculated for thermal denaturation of calorimetric domain II. Since the ATPase site is localized in the motor domain of the S1 molecule (Figure 5), these results, in good agreement with those obtained from experiments on S1 intrinsic tryptophan fluorescence (Figures 7 and 8), are in favor of correspondence of calorimetric domain II to the motor domain of S1.

### 3.4. Heat-Induced Aggregation of S1 Isoforms

The irreversible thermal denaturation of S1 is accompanied by its aggregation [16,20]. We applied DLS to analyze the thermally induced aggregation of S1 isoforms and to investigate in detail the mechanism of this process. Previous studies have shown that DLS allows determining the size of particles formed in the process of protein aggregation during the heating [25–27]. Using this method, we studied heat-induced aggregation of S1(A1) and S1(A2) in the presence of 100 mM KCl.

**Figure 11.**
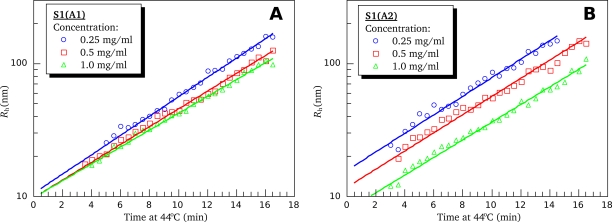
Time course of S1(A1) (**A**) and S1(A2) (**B**) aggregation upon heating at 44 °C at different protein concentrations as measured by growth of mean hydrodynamic radius (*R*_h_). All experiments were performed in presence of 100 mM KCl. Distribution of the hydrodynamic radius by size was monomodal during all the period of observation.

**Figure 12.**
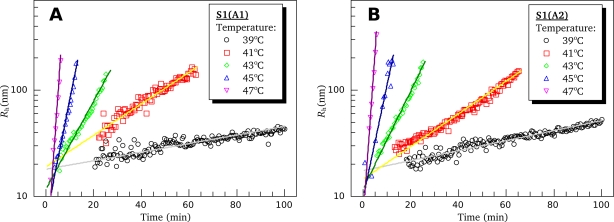
Time course of S1(A1) (**A**) and S1(A2) (**B**) aggregation upon heating at different temperatures as measured by growth of the mean hydrodynamic radius (*R*_h_). All experiments were performed in presence of 100 mM KCl. Protein concentration was constant and equal to 0.5 mg/mL for both S1 isoforms. Distribution of the hydrodynamic radius was monomodal during all the period of observation.

**Figure 13.**
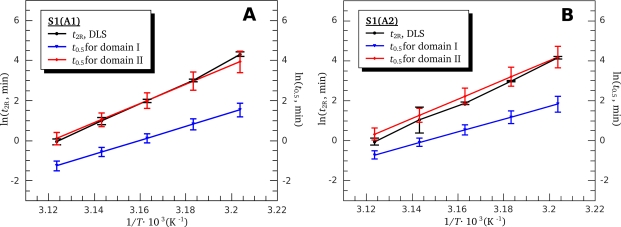
Comparison of the DLS data on the thermally induced aggregation of S1(A1) (**A**) and S1(A2) (**B**) (the temperature dependences of the *t*_2R_ values shown by black lines) with the DSC data on the thermal denaturation of calorimetric domains I and II (the temperature dependences of the *t*_0.5_ values shown by blue and red lines for domains I and II, respectively). The *t*_0.5_ values were calculated from the data presented in Table 1 (in the case of domain II, the *t*_0.5_ values for the first stage of its denaturation are only presented).

Unlike thermal denaturation, aggregation of proteins is always a reaction of interaction between two (or more than two) proteins molecules. Hence, at least one of its stages should be the second-order (or higher order) reaction. In other words, one can expect that, in general, aggregation of proteins should depend on their concentration. Nevertheless, our preliminary DLS results obtained in the presence of 100 mM KCl with mixed (not separated into isoforms) S1 preparation have demonstrated that under these conditions the heat-induced aggregation of S1, as measured by an increase in the hydrodynamic radius (*R*_h_) of the formed aggregates, did not depend on the S1 concentration within a rather wide range, from 0.125 to 2.0 mg/mL (data not shown). This effect was also observed with separate S1 isoforms. Figure 11 shows, in semi-logarithmic coordinates, the typical dependences of *R*_h_ value for both S1 isoforms on the time of their incubation at 44 °C. These dependences are well described by the following exponents:

(26) $$R_{\text{h}}=R_{\text{h}}^{0}\cdot \text{exp}\left[\frac{\text{ln}(2)}{t_{2\text{R}}}\cdot (t-t_{0})\right],\;\;\;\;\;\text{if}\;\;\;t>t_{0}$$

where *t*_0_ is the delay time before the beginning of *R_h_* growth (*i.e*., lag period of the aggregation), $R_{\text{h}}^{0}$ is the initial hydrodynamic radius (at *t* = *t*_0_), and *t*_2R_ is the time interval over which the hydrodynamic radius of the protein aggregates increases twofold. The parameter *t*_2R_ characterizes the rate of aggregation [27]. The higher the *t*_2R_ value, the lower is the rate of aggregation. One can see that straight lines obtained at different protein concentrations are in parallel and differ from each other only by shift along the abscissa axis (Figure 11). The observed shift of the lines is suggested to be due to some uncertainty caused by preliminary heating of the protein solution in the instrument from storage temperature (4 °C) to 44 °C, *i.e.*, the temperature, at which the kinetics of *R*_h_ growth was recorded. We performed these experiments several times and only the slope of the lines was reproducible but not their absolute location on the abscissa axis. The fact that lines in both Figure 11A and Figure 11B have the same slope indicates that the parameter *t*_2R_ is the same for all the concentrations studied. Optimal values of this parameter have been found as described in Experimental Section (see subsection 4.7). Within the error limit (confidence interval of 95%), these parameters were identical at all protein concentrations and equal to 4.5 ± 0.4 min for both S1 isoforms. Hence, we can conclude that the S1 aggregation is limited by some first-order reaction. For better understanding the mechanism of S1 thermal aggregation, we investigated the dependence of the *t*_2R_ value on temperature. Figure 12 shows, in semi-logarithmic coordinates, the dependences of *R*_h_ value for the both S1 isoforms on the time of their incubation at different temperatures. It is clearly seen that the rate of aggregation increases upon the increase in the temperature. At all temperatures studied, the kinetic curves of *R*_h_ growth are well described by Equation 26, thus allowing us to calculate the *t*_2R_ values at different temperatures. The temperature dependences of the *t*_2R_ values for both S1 isoforms are presented on Figure 13 (black lines). These dependences are compared with those of the half-life values

(27) $$t_{0.5}=\frac{\text{ln}(2)}{k}$$

(where *k* is the first-order rate constant) calculated from the data presented in Table 1 for calorimetric domains I and II (Figure 13, blue and red lines, respectively). It is clearly seen that for both S1 isoforms the *t*_2R_ values coincide with the *t*_0.5_ values calculated for the first stage of thermal denaturation of calorimetric domain II, which was identified above as the motor domain of the S1 molecule.

We also calculated the parameters *E_a_* and T^*^ of the Arrhenius equation (Equation 2) for the *R*_h_ growth process of S1 aggregates. The *E_a_* value was equal to 430 ± 50 kJ/mole for S1(A1) and 440 ± 70 kJ/mole for S1(A2), and T^*^ was equal to 320.8 ± 0.6 K for both S1 isoforms. It is seen that both these parameters agree with those obtained from DSC experiments for the first stage of denaturation of calorimetric domain II (see Table 1). Thus, just this reaction, *i.e.*, the first stage of denaturation of calorimetric domain II, limits the overall process of S1 thermal aggregation under high ionic strength conditions (in the presence of 100 mM KCl).

**Figure 14.**
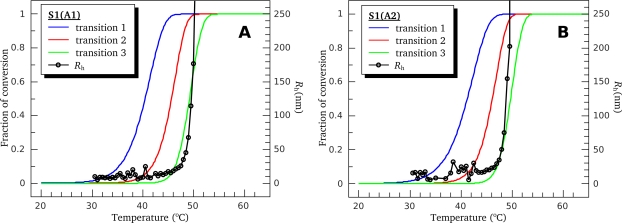
Temperature dependences of the *R*_h_ growth obtained from the DLS experiments for S1(A1) (**A**) and S1(A2) (**B**) (black circles and lines) in comparison with fraction of conversion for calorimetric transitions 1, 2, and 3 obtained from the DSC experiments on these S1 isoforms and calculated as in Figure 8 (colored curves). Heating rate was 1 °C/min in both cases. Proteins concentration in the DLS experiments was 0.5 mg/mL.

For better understanding of the delay in the aggregation process (*i.e.*, the nature of the lag period expressed by the *t*_0_ value), we also investigated the temperature dependences of *R*_h_ growth upon heating the S1 isoforms with constant rate of 1 °C/min under high ionic strength conditions (in the presence of 100 mM KCl). These dependences were the same for both S1 isoforms and independent of the protein concentration within the range from 0.25 to 1.0 mg/mL up to *R*_h_ values of 3000 nm (data not shown). Figure 14 shows initial parts of the temperature dependences of *R*_h_ growth (up to 250 nm) in comparison with fractions of conversion for calorimetric transitions 1, 2, and 3 obtained from DSC experiments (these curves were calculated as it was described in the legend to Figure 8). It is clearly seen that for both S1(A1) and S1(A2) a few degrees delay is observed between the process of *R*_h_ fast growth and the first stage of thermal denaturation of the calorimetric domain II (transition 2) that limits the overall aggregation process. This observation leads to the conclusion that nucleation (*i.e.*, formation of start aggregates that are seeds for following aggregation [27]) is an important stage of the overall process of the S1 thermal aggregation. Obviously, formation of necessary amount of these seeds needs some time, which accounts for the observed delay in the aggregation process.

A unique feature of the S1 thermal denaturation is a “blue shift” of its intrinsic fluorescence spectrum (Figure 6). This spectral shift means that the environment of Trp residues becomes more hydrophobic after denaturation. In this respect, S1 is quite different from most of other proteins that usually demonstrate a “red shift” (*i.e.*, the shift of the spectrum to higher wavelengths). The question arises whether the blue shift is due to S1 irreversible thermal denaturation or aggregation that accompanies the denaturation process. To answer the question we have compared temperature induced changes of S1 fluorescence (Figure 8) with its aggregation (Figure 14). We have shown that thermal aggregation of S1 is limited by the first stage of denaturation of calorimetric domain II (that corresponds to calorimetric transition 2). At the same time, as it is clearly seen from Figure 14, there is a several degrees lag between calorimetric transition 2 (red curve) and the growth of average hydrodynamic radius of S1 aggregates (black points and curve in Figure 14). Let us assume that thermal denaturation of S1 is accompanied by exposure of Trp residues and the observed blue shift of intrinsic Trp fluorescence spectrum is due to subsequent aggregation of fully or partially denatured (or unfolded) protein molecules that lead to burial of Trp residues in hydrophobic aggregate core. If the assumption is correct we expect the appearance of red shift of intrinsic fluorescence spectrum in the temperature interval between the calorimetric transition 2 (red curve in Figures 8 and 14) and the process of *R*_h_ fast growth (black points and curve in Figure 14), and the replacement of the red shift by the blue shift at higher temperature region. Thus, the temperature dependence of parameter *A* would be non-monotone. However, it is clearly seen from Figure 8 that parameter *A* grows with temperature monotonely. Moreover, the changes in parameter *A* occur in the temperature interval between calorimetric transitions 2 and 3, so slightly outstrip the growth of the aggregates. Despite the fact that described investigations of temperature dependences of S1 fluorescent properties and its thermal aggregation were carried out at different protein concentration (see Experimental Section), we suggest that the comparison of intrinsic fluorescence data and DLS data is reasonable. We observed that pronounced aggregation of both S1 isoforms occurred in the same temperature intervals for both high and low (0.05 mg/mL, as in the fluorescence studies; data not shown) S1 concentrations. Moreover, the earlier investigations revealed that the blue shift of S1 intrinsic fluorescence spectrum also takes place at protein concentration of 0.5 mg/mL and it also has a monotonic character [16]. Therefore, we have to conclude that observed blue shift of S1 intrinsic fluorescence spectrum is due to changes in the protein molecule occurring in the process of its irreversible thermal denaturation but not its aggregation.

Based on the data obtained, we can propose the following mechanism of S1 thermal aggregation. The first stage of this process is nucleation, when several partially or fully denatured S1 molecules stick together and form the primary aggregates (so-called start aggregates [27]). The experiments on thermal aggregation of the proteins showed that the size of the start aggregates remained unchanged at variation of the initial concentration of a protein [25–27]. This means that the concentration of the start aggregates formed at the initial stages of the aggregation process varies in direct proportion with the initial concentration of a protein. The time needed for formation of critical number of the start aggregates is the parameter *t*_0_ in the Equation 26. The following growth of the start aggregates is realized by the attachment of the individual S1 molecules, whose motor domains are on the stage of kinetic intermediate (see scheme 19), to these aggregates. It should be noted that the rate of this sticking process is much higher than the rate of the first stage of thermal denaturation of the S1 motor domain, which is the rate-limiting step for the whole process of the growth of aggregates. Further denaturation of the motor domain (*i.e.*, the second stage of this process) probably occurs after inclusion of such partially denatured S1 molecules into the aggregates.

The above proposed mechanism of S1 thermal aggregation disagrees with earlier proposed scheme of heat-induced aggregation of proteins [27], according to which the start aggregates interact with each other resulting in formation of the amorphous aggregates. It seems highly unlikely that this scheme is applicable in the case of S1 thermal aggregation. If the S1 aggregation process occurred due to interactions between start aggregates, one would have supposed that at all studied protein concentrations these aggregates are formed from denatured S1 molecules and stick with each other much faster than denaturation of the S1 motor domain occurs, whose first stage is the rate-limiting reaction for the overall process. In other words, this would be represented as follows: protein molecules slowly denature and then rapidly stick together into start aggregates, which, in turn, very rapidly interact with each other to form amorphous aggregates. This mechanism seems to be highly improbable. Furthermore, this scheme becomes too complicated and ungrounded in the case of S1 aggregation, because of involving the experimentally uncorroborated stage of interaction between start aggregates, which is separated from the stage of their formation. In contrast, the mechanism proposed here, according to which the growth of the start aggregates is accounted for by the attachment of the individual S1 molecules to these aggregates, is devoid of these shortcomings.

It should be noted that the earlier proposed model of heat-induced aggregation of proteins based on the interaction between start aggregates [27] can explain very successfully the aggregation of many different proteins and enzymes. However, the results of the present work evidence that thermally induced aggregation of myosin S1 cannot be explained by this model and some new mechanism is needed to explain these data. This mechanism, which is described above for the first time, is a new promising approach for analyzing amorphous thermal aggregation of proteins.

## 4. Experimental Section

### 4.1. Protein Preparations

S1 from rabbit skeletal myosin was prepared by digestion of myosin filaments with TLCK-treated *α*-chymotrypsin [4]. The concentration of S1 was estimated spectrophotometrically using extinction coefficient E^1%^ at 280 nm of 7.5 cm^−1^. S1 preparation was separated into S1(A1) and S1(A2) isoforms by means of ion exchange chromatography on a column of SP-trisacryl [28]. All proteins were homogeneous according to SDS-PAGE [29]. Both preparations of S1 isoforms were homogeneous and no proteolysis of either heavy or light chains was observed.

### 4.2. Differential Scanning Calorimetry (DSC)

DSC experiments were performed on a DASM-4M differential scanning microcalorimeter (Institute for Biological Instrumentation, Pushchino, Russia) as described earlier [30–32]. Samples containing S1 isoforms (1.2–1.9 mg/mL) were heated with constant rate of 1 °C/min from 15 °C to 85 °C in 20 mM Hepes-KOH (pH 7.3) containing 1 mM MgCl_2_ in the presence or absence of 100 mM KCl. In order to check the reversibility of thermal denaturation after the first scan and subsequent cooling, the protein samples were reheated. Thermal denaturation of both S1 isoforms was fully irreversible. Calorimetric traces were corrected for instrumental background using special DSC approach described earlier [33]. Briefly, DSC measurements were performed not only in the usual way, when the protein was placed into the sample cell and the buffer was placed into the reference cell, but also vice versa, with the same protein sample in the reference cell and the buffer in the sample cell. This inverted curve was then subtracted from the curve obtained by the usual way. The above DSC approach allowed us to subtract the instrumental baseline with very high precision [33]. Annealing procedures were performed by pre-heating the protein samples at appropriate temperature for definite period of time just before the DSC measurements (the temperature and time for these procedures were calculated as described in Section 2). All calorimetric traces were subjected to time response correction as described in [34] before the further analysis. Chemical base line correction was performed using the approach described below.

### 4.3. Estimation of Chemical Base Line in DSC Experiments

The thermogram for a protein undergoing one-stage reversible as well as one-stage irreversible denaturation may be described by the following equation

(28) $$C_{\text{p}}(T)=C_{\text{p}}^{N}(T)\cdot (1-\xi (T))+C_{\text{p}}^{D}(T)\cdot \xi (T)+\Delta H\cdot \frac{d\xi }{dT}$$

where ξ(*T*) is the degree of completeness of the transition (the portion of the denatured state), Δ*H* is the change of enthalpy when passing from the native state to the denatured state (it is assumed that Δ*H* is independent of the temperature), $C_{\text{p}}^{N}(T)$ and $C_{\text{p}}^{D}(T)$ are heat capacity of the native and denatured states, respectively. On the relatively small intervals of temperature the functions $C_{\text{p}}^{N}(T)$ and $C_{\text{p}}^{D}(T)$ may be considered as linear functions of temperature with rather high accuracy (strictly speaking, polynomial functions should be used). In our investigations only last term of Equation 28 is of immediate interest. This term is called excess heat capacity or excess heat absorption:

(29) $$C_{\text{p}}^{\text{ex}}(T)=\Delta H\cdot \frac{d\xi }{dT}$$

The other terms in the sum are called the chemical base line. We are now faced with the problem of the subtracting of the chemical base line. In the case of one-stage transition the chemical base line as a function of temperature may be easily estimated by the numerical solution of the differential equation 28 for ξ after the determination of the functions $C_{\text{p}}^{N}(T)$ and $C_{\text{p}}^{D}(T)$. These functions are estimated by the linear approximation of the parts of the thermogram located immediately before and right after the peak, respectively.

In the case of the multidomain protein or protein with several thermal transitions the thermogram is an algebraic sum of the equations of the type 28:

(30) $$C_{\text{p}}(T)=\sum_{i}C_{\text{p},\;\;i}^{N}(T)+\sum_{i}\Delta C_{\text{p},\;\;i}(T)\cdot \xi_{i}(T)+\sum_{i}\Delta H_{i}\cdot \frac{d\xi_{i}}{dT}$$

where $\Delta C_{\text{p},\;\;i}(T)=C_{\text{p},\;\;i}^{D}(T)-C_{\text{p},\;\;i}^{N}(T)$ is the difference between the heat capacities of the denatured and native states of the *i*-th transition. In this case the excess heat capacity is equal to

(31) $$C_{\text{p}}^{\text{ex}}(T)=\sum_{i}\Delta H_{i}\cdot \frac{d\xi_{i}}{dT}$$

and its estimation becomes a non-trivial problem (strictly speaking, this is an insoluble problem in general). The reason is that each transition superinduces at least two independent parameters (the polynomial coefficients of Δ*C_p, i_*(*T*)). Besides, to decompose Equation 30 into its components namely the equations of a type of Equation 28, the mechanism of thermal denaturation should be established. To work out this problem, we need the function $C_{\text{p}}^{\text{ex}}(T)$ to be known.

As a null approximation the approach which is close to that described by Filimonov *et al*. [35] may be used. Let us assume that the contribution of each transition in the heat capacity jump is proportional to the contribution of enthalpy of this transition in the overall enthalpy:

(32) $$\frac{\Delta C_{\text{p},\;\;i}(T)}{\sum_{i}\Delta C_{\text{p},\;\;i}(T)}=\frac{\Delta H_{i}}{\sum_{i}\Delta H_{i}}$$

This assumption means that the ratio of each polynomial coefficient of Δ*C_p, i_*(*T*) to the corresponding coefficient of ∑*_i_*Δ*C_p, i_*(*T*) are identical and equal to the ratio Δ*H_i_*/∑*_i_*Δ*H_i_*. The above-mentioned assumption has a definite sense. Actually, the more bonds are broken during a given transition, the higher is the contribution of this transition in the overall enthalpy of a whole process. However, the more broken bonds, the more new degrees of freedom appear and, correspondingly, the higher is the heat capacity jump. It should be noted, however, that this relationship may be not linear.

Substitute Equation 32 in Equation 30:

(33) $$C_{p}(T)=\sum_{i}C_{\text{p},\;\;i}^{N}(T)+\sum_{i}\left(\frac{\Delta H_{i}\cdot \underset{i}{\Sigma }\Delta C_{\text{p},\;\;i}(T)}{\underset{i}{\Sigma }\Delta H_{i}}\cdot \xi_{i}(T)\right)+\sum_{i}\Delta H_{i}\cdot \left(\frac{\underset{i}{\Sigma }\Delta H_{i}\cdot \frac{d\xi_{i}}{dT}}{\underset{i}{\Sigma }\Delta H_{i}}\right)$$

The following designations are used:

(34) $$\Delta H=\sum_{i}\Delta H_{i},\;\;\;\;\Delta C_{\text{p}}=\sum_{i}\Delta C_{\text{p},\;\;\;i},\;\;\;\;\;\;\;C_{\text{p}}^{N}=\sum_{i}C_{\text{p},\;\;\;i}^{N}$$

After substitution of these designations in Equation 33 we obtain the following equation:

(35) $$C_{p}(T)=C_{\text{p}}^{N}(T)+\Delta C_{\text{p}}(T)\cdot \sum_{i}\frac{\Delta H_{i}\cdot \xi_{i}(T)}{\Delta H}+\Delta H\cdot \frac{d}{\text{dT}}\left(\sum_{i}\frac{\Delta H_{i}\cdot \xi_{i}(T)}{\Delta H}\right)$$

Formally this equation coincides with Equation 28 accurate to

$$\xi =\sum_{i}\frac{\Delta H_{i}\cdot \xi_{i}(T)}{\Delta H}$$

Thus, to estimate the chemical base line of the thermogram of protein with several transitions, we can use the same approach as in the case of a single transition. Strictly speaking, assumption 32 is correct only in the null approximation. However, since $C_{\text{p}}^{\text{ex}}$ usually exceeds several times in amplitude the Δ*C*_p_ value, the inaccuracy introduced in the $C_{\text{p}}^{\text{ex}}(T)$ value by this approximation should be not high.

On this basis, the estimation of the chemical base line and its subtraction may be carried out as follows. We select four points on the experimental dependence of heat absorption *C*_p_(*T*). The first point (*T*_1_) corresponds to the beginning of the linear part of the thermogram before the peak of excess heat absorption, the second point (*T*_2_) corresponds to the beginning of the peak and simultaneously the end of the linear part before the peak, the third point (*T*_3_) corresponds to the end of the peak and the beginning of the linear part after the peak and the fourth point (*T*_4_) corresponds to the end of the linear part after the peak. The linear part before the peak is taken as $C_{\text{p}}^{N}(T)$. The linear part after the peak is taken as $C_{\text{p}}^{D}(T)$. Further we determine the corresponding coefficients of the linear approximation of $C_{\text{p}}^{N}(T)$ and $C_{\text{p}}^{D}(T)$. The obtained coefficients are used for the calculation of $C_{\text{p}}^{N}(T)$ and $C_{\text{p}}^{D}(T)$ in the temperature interval form first to fourth point (the calculated values of these functions are designated as $C_{\text{p},\;\text{calc}}^{N}(T)$ and $C_{\text{p},\;\text{calc}}^{D}(T)$, respectively). As a null approximation the chemical base line (designated as *C*_CBL_(*T*)) is taken equal to $C_{\text{p},\;\text{calc}}^{N}(T)$ in the interval from *T*_1_ to *T*_2_. In the interval from *T*_3_ to *T*_4_ the chemical base line is taken equal to $C_{\text{p},\;\text{calc}}^{D}(T)$. As for the interval from *T*_2_ to *T*_3_, the chemical base line is assumed to be the straight line connecting the second and third points on the experimental curve of heat absorption. Then we estimate the null approximations for Δ*H* and ξ(*T*):

(36) $$\Delta H=\int_{T_{1}}^{T_{4}}(C_{\text{p}}(T)-C_{\text{CBL}}(T))\;\;\;dT,\;\;\;\;\;\xi (T)=\frac{1}{\Delta H}\cdot \int_{T_{1}}^{T}(C_{\text{p}}(T)-C_{\text{CBL}}(T))\;dT$$

After that, the excess heat capacity in the interval from *T*_1_ to *T*_4_ is calculated by the formula:

(37) $$C_{\text{p}}^{\text{ex}}(T)=C_{\text{p}}(T)-C_{\text{p},\;\text{calc}}^{N}(T)\cdot (1-\xi (T))-C_{\text{p},\;\text{calc}}^{D}(T)\cdot \xi (T)$$

The calculated excess heat capacity is used for estimation of Δ*H* and ξ(*T*). From the newly found function ξ(*T*) we calculate the corrected function $C_{\text{p}}^{\text{ex}}(T)$. The procedure is repeated as long as the difference between the values of Δ*H* calculated on *i*-th and (*i* – 1)-th step is not less than a definite small quantity (for example, 0.01% from Δ*H*).

The proposed method of the estimation of the chemical base line is close to the algorithm elaborated by Filimonov *et al*. [35]. The main difference between two methods is the following. Filimonov *et al*. assumed that Δ*C_p, i_* was independent of temperature. In our opinion, this approximation is too rough. This was the reason for the elaboration of a new modification of the method for the estimation of the chemical base line.

### 4.4. Intrinsic Fluorescence

Fluorescence studies were performed on a Cary Eclipse spectrofluorimeter (Varian) equipped with a Peltier-controlled cell holder and thermoprobes. Intrinsic tryptophan fluorescence was measured at protein concentration of 0.05 mg/mL in 20 mM Hepes-KOH (pH 7.3) containing 1 mM MgCl_2_ in the presence or absence of 100 mM KCl. Fluorescence was excited at 297 nm (slit width 5 nm) and recorded in the range of 310–395 nm (slit width 2.5 nm). The proteins samples were heated with constant rate of 1 °C/min from 20 °C to 70 °C, and the fluorescence intensities at 320 nm and 365 nm were recorded. The position and form of the fluorescence spectra were characterized by parameter *A* (see Equation 24) [36–38]. In order to qualitatively analyze the temperature dependence of intrinsic fluorescence we used the fluorescence phase plots (the dependence of fluorescence intensity at 365 nm on the intensity of fluorescence at 320 nm obtained at different temperatures) [23].

### 4.5. ATPase Inactivation

The thermally induced inactivation of S1 isoforms’ ATPase was measured after heating the protein (0.5 mg/mL) at constant temperature in the medium containing 20 mM Hepes (pH 7.3), 1 mM MgCl_2_ and 100 mM KCl. S1(A1) or S1(A2) aliquots were heated at appropriate temperature for appropriate periods of time, then cooled and subjected to ATPase measurements. Experiments were performed at different temperatures within the range from 39 °C to 47 °C. The ATPase activity of S1 isoforms (K^+^-EDTA-ATPase) was determined by P_i_ release [39] at 25 °C in the medium containing S1 (0.04 mg/mL), 1 mM ATP, 0.5 M KCl, 5 mM EDTA and 50 mM Tris-HCl (pH 7.5). Reaction was initiated by addition of ATP and stopped after 10 min of incubation by addition of HClO_4_ to final concentration of 2.5%.

### 4.6. Dynamic Light Scattering (DLS)

DLS measurements were performed on a Photocor Complex apparatus (Photocor Instruments Inc., USA) equipped with a temperature controller. The sample was illuminated by a 632.8 nm laser light, and the scattering signal was observed at an angle of 90°. DLS data were accumulated and analyzed with multifunctional real-time correlator Photocor-FC. DynaLS software (Alango, Israel) was used for polydisperse analysis of DLS data. The kinetics of S1 isoforms aggregation was studied by measuring an increase in the mean hydrodynamic radius of the particles (*R*_h_) upon incubation of S1 in 20 mM Hepes (pH 7.3) containing 1 mM MgCl_2_ and 100 mM KCl. The kinetics of S1 isoforms’ aggregation was studied either at constant temperature of 44 °C and different protein concentrations, from 0.25 to 1.0 mg/mL, or at constant protein concentration of 0.5 mg/mL and different temperatures, from 39 °C to 47 °C. Temperature dependences of *R*_h_ growth were studied upon heating of S1 isoforms’ preparations with constant rate of 1 °C/min in the same buffer as in the kinetic experiments.

### 4.7. Calculation Procedures

The algorithms for estimation of chemical base line, calculation of *W*(*T*) function and appropriate annealing conditions (time and temperature) were realized as scripts running in the Matlab (The MathWorks, Inc.) environment. The optimization procedures used for calculation of en-route and final values of calorimetric domains’ parameters were also performed in the Matlab environment. For this purpose, we have written scripts-wrappers for the lsqcurvefit function of the Optimization Toolbox for Matlab.

Optimization algorithms (embedded into the Optimization Toolbox) allow to calculate only optimal values of the parameters, but not their confidence intervals. Moreover, because of specific features of the calorimetric experiment, the systematic error exceeds many times the random error. This renders the use of regression statistics for estimating the confidence intervals impossible. For this reason we used the following (rather rough) approach to estimate the error in the values of the calorimetric parameters. We carried out a number of different optimization procedures in the process of calculation of the values of these parameters. In one case, all the experimental $C_{\text{p}}^{\text{ex}}$ curves obtained for the given S1 preparation (the curve obtained in the experiment without preliminary annealing and two curves obtained for the preparations subjected to two different annealing procedures before the calorimetric experiment) were simultaneously used as the input data. The values of the calorimetric parameters obtained after such kind of optimization were admitted to be final and presented in Table 1. We also performed several other optimization procedures using as an input data only one $C_{\text{p}}^{\text{ex}}$ curve (obtained in an experiment with or without preliminary annealing) per procedure. Different optimization procedures produced different values of the calorimetric parameters. However, the difference in *E_a_* and T^*^ values calculated for the same transition of the same S1 preparation never exceeded 40 kJ/mole and 0.4 K, respectively. The main source of the enthalpy error is inexact subtraction of chemical base line. Selection of different linear segments on thermograms before and after the peak of excess heat absorption results in slightly different values of calorimetric enthalpy. In our experiments this difference never exceeded 10%. Thus, we assumed the following error values for the calorimetric parameters presented: 0.4 K for T^*^, 40 kJ/mole for *E_a_* and 10% of the value for Δ*H*.

The optimal values of the rate constants for the heat-induced S1 ATPase inactivation and the optimal values of the *t*_2R_ parameters for the S1 aggregates growth were found with 95% confidence interval, using the Trust-Region algorithm embedded into the Curve Fitting Toolbox in the Matlab environment.

The values of the rate constants and half-life times of the S1 calorimetric domains were calculated using Arrhenius equation (Equation 2) and corresponding *E*_a_ and T^*^ values given in Table 1. The errors of these rate constants and half-life times were calculated as indirect errors caused by the errors in calculated calorimetric parameters (shown in Table 1) and by the error in incubation temperature that was assumed to be equal to 0.5 °C.

The optimal values of the parameters of Arrhenius equation (2) for the process of *R*_h_ growth were also estimated using the Trust-Region algorithm embedded into the Curve Fitting Toolbox in the Matlab environment. The errors of these parameters were calculated as indirect errors caused by the errors in calculated values of the *t*_2R_ parameters and by the error in incubation temperature that was assumed to be equal to 0.5 °C.

## 5. Conclusions

In the present study we have found no appreciable difference between S1(A1) and S1(A2) in their aggregation properties under ionic strength conditions close to those in the muscle fiber (in the presence of 100 mM KCl). This means that under these conditions the presence of additional *N*-terminal segment in myosin A1 light chain does not affect the aggregation properties of S1. Furthermore, under these conditions S1 thermal aggregation follows its thermal denaturation and the aggregation rate is limited by irreversible denaturation of the motor domain of S1 molecule. From these data a new mechanism describing the amorphous thermal aggregation of proteins has been proposed, which explains specific aggregation properties of S1. According to this mechanism, the first stage of the aggregation process, nucleation, is directly followed by sticking denatured S1 molecules to these start aggregates, but not by interactions between the start aggregates.

Some difference between the two S1 isoforms was only revealed by DSC, independently of the ionic strength conditions, in the thermal denaturation of the regulatory domain. This is, however, quite an expected effect as the alkali light chains are associated with this domain of the S1 molecule. Thus, the interaction of the *N*-terminal extension of the A1 light chain with the motor domain [12–14] seems to be too weak to produce any effect on its thermal denaturation even at low ionic strength.

A noticeable influence of the A1 *N*-terminal segment on the S1 thermal aggregation was observed only at relatively low ionic strength [15,16,40]. Under these conditions the intermolecular interactions of the A1 *N*-terminal extension appear to be the main factor underlying the aggregation properties of S1. In contrast, the results of the present study indicate that at relatively high ionic strength (close to ionic strength conditions in muscle fibers) these interactions are too weak to have any effect on the S1 thermal aggregation.

## Figures and Tables

**Table 1.** The results of the fitting of the DSC data for S1 preparations after final optimization in the framework of the two-domain model.

**High ionic strength conditions (100 mM KCl)**						
**Parameter**	Domain I (transition 1)		Domain II (transition 2)		Domain II (transition 3)	
	S1(A1)	S1(A2)	S1(A1)	S1(A2)	S1(A1)	S1(A2)
T^*^, K	317.5 *±* 0.4	319.0 *±* 0.4	321.2 *±* 0.4	321.6 *±* 0.4	324.5 *±* 0.4	324.7 *±* 0.4
*E_a_*, kJ/mole	290 *±* 40	265 *±* 40	400 *±* 40	400 *±* 40	340 *±* 40	380 *±* 40
Δ*H*, kJ/mole	200 *±* 20	200 *±* 20	1030 *±* 100	970 *±* 100	500 *±* 50	300 *±* 30

**Low ionic strength conditions (no KCl)**						
**Parameter**	Domain I (transition 1)		Domain II (transition 2)		Domain II (transition 3)	
	S1(A1)	S1(A2)	S1(A1)	S1(A2)	S1(A1)	S1(A2)
T^*^, K	319.2 *±* 0.4	321.4 *±* 0.4	323.0 *±* 0.4	323.0 *±* 0.4	325.5 *±* 0.4	325.6 *±* 0.4
*E_a_*, kJ/mole	230 *±* 40	250 *±* 40	350 *±* 40	400 *±* 40	390 *±* 40	390 *±* 40
Δ*H*, kJ/mole	370 *±* 40	380 *±* 40	1260 *±* 130	720 *±* 70	400 *±* 40	260 *±* 30
